# Evidence of Nonlinear Seismic Effects in the Earth from Downhole Distributed Acoustic Sensors

**DOI:** 10.3390/s22239382

**Published:** 2022-12-01

**Authors:** Alexey Yurikov, Boris Gurevich, Konstantin Tertyshnikov, Maxim Lebedev, Roman Isaenkov, Evgenii Sidenko, Sinem Yavuz, Stanislav Glubokovskikh, Valeriya Shulakova, Barry Freifeld, Julia Correa, Todd J. Wood, Igor A. Beresnev, Roman Pevzner

**Affiliations:** 1 Centre for Exploration Geophysics, Curtin University, GPO Box U1987, Perth, WA 6845, Australia; 2 Lawrence Berkeley National Laboratory, 1 Cyclotron Road, Berkeley, CA 94720, USA; 3 CSIRO, Australian Resources Research Centre, 26 Dick Perry Avenue, Kensington, WA 6151, Australia; 4 Class VI Solutions, Inc., 711 Jean Street, Oakland, CA 94610, USA; 5 Department of Geological and Atmospheric Sciences, Iowa State University, 253 Science I, 2237 Osborn Dr., Ames, IA 50011, USA

**Keywords:** nonlinear seismology, optical fibers, distributed acoustic sensing, heterodyne, downhole

## Abstract

Seismic velocities and elastic moduli of rocks are known to vary significantly with applied stress, which indicates that these materials exhibit nonlinear elasticity. Monochromatic waves in nonlinear elastic media are known to generate higher harmonics and combinational frequencies. Such effects have the potential to be used for broadening the frequency band of seismic sources, characterization of the subsurface, and safety monitoring of civil engineering infrastructure. However, knowledge on nonlinear seismic effects is still scarce, which impedes the development of their practical applications. To explore the potential of nonlinear seismology, we performed three experiments: two in the field and one in the laboratory. The first field experiment used two vibroseis sources generating signals with two different monochromatic frequencies. The second field experiment used a surface orbital vibrator with two eccentric motors working at different frequencies. In both experiments, the generated wavefield was recorded in a borehole using a fiber-optic distributed acoustic sensing cable. Both experiments showed combinational frequencies, harmonics, and other intermodulation products of the fundamental frequencies both on the surface and at depth. Laboratory experiments replicated the setup of the field test with vibroseis sources and showed similar nonlinear combinations of fundamental frequencies. Amplitudes of the nonlinear signals observed in the laboratory showed variation with the saturating fluid. These results confirm that nonlinear components of the wavefield propagate as body waves, are likely to generate within rock formations, and can be potentially used for reservoir fluid characterization.

## 1. Introduction

Exploration seismology usually assumes that the earth is a linearly elastic medium, i.e., the relationship between stress and strain is linear. This implies that the stiffness of the medium (the inverse of the derivative of the strain with respect to stress) is independent of stress. However, many experiments show the significant stress dependency of elastic-wave velocities and elastic moduli; this stress sensitivity is usually attributed to the heterogeneous microstructure of rocks and presence of highly compliant voids at the contact between solid particles [1,2]. This means that the relationship between stress and strain in rocks is nonlinear. A range of geophysical experiments have demonstrated nonlinear wave effects in the earth [3,4,5,6], which result in the generation of higher harmonics and combinational frequencies of the transmitted and interacting seismic waves. 

The process of the generation of combinational frequencies of two signals mixed in a nonlinear medium was first used in communication engineering and is known as heterodyning [7]. Heterodyning can be applied to seismic exploration as well. Theoretically, it can be used for broadening the frequency band of the seismic signal, especially in the low-frequency range. Vibroseis sources can produce a seismic signal in a limited frequency band, typically 5–200 Hz. Extending this band to lower frequencies using a combinational frequency *f*_1_ − *f*_2_ can potentially improve resolution of seismic imaging. Over the last four decades, a number of studies [3,6,8,9] reported a heterodyning effect produced by two groups of surface seismic vibrators and recorded by surface geophones; see also patents [10,11]. However, discussion and analysis of the application of these technologies is rather limited.

Another application of nonlinear seismic effects is in the monitoring and characterization of subsurface properties using components of a wavefield generated as the result of nonlinear wave interaction in rocks. A range of laboratory measurements have confirmed that heterogeneous media such as rocks are nonlinear, and the magnitude of the nonlinearity is two to three orders higher than in homogeneous solids [12,13]. In a field experiment, Zhukov et al. [14] recorded reflected waves carrying combinational frequencies by a line of surface receivers when two groups of vibrators separated by the distance of 5400 m simultaneously generated a 30–100 Hz sweep and a single modulation frequency of 22 Hz. Zhukov et al. [14] processed nonlinear signals by correlating the recorded data with the differential sweep (i.e., the sweep shifted in a frequency domain by the value of the modulation frequency). They reported that the intensity of the reflected nonlinear signal varied along the 2D seismic profile and was the strongest in the formations saturated with hydrocarbons. Similar results were reported by Shulakova [15].

Kuvshinov et al. [16] argued that a correlation procedure employed by Zhukov, et al. [14] may produce artefacts because the frequency spectrum of the nonlinearly generated waves would not coincide with the shifted frequency spectrum of the source sweep. A broader point of many field studies on nonlinear effects is that most of the nonlinear signal is likely to be generated in the shallow subsurface [17]. Moreover, Kuvshinov et al. [16] modelled nonlinear signals from deep formations and showed that their amplitudes at the surface were typically below the seismic noise level and could not be measured at the existing level of technology.

Among technological developments required to advance the study of nonlinear seismic effects, one was already available decades ago. Vertical seismic profiling (VSP) offers an opportunity to place seismic receivers in a well deep inside rock formations and analyze direct waves, which have higher amplitude than the reflected waves studied previously. Campman et al. [18] reported experiments where the surface receivers were complemented by downhole geophones to record nonlinear components of the wavefield generated by two vibrators producing monochromatic signals of 33 and 45 Hz. Campman et al. [18] observed that nonlinear harmonics attenuated with depth faster than fundamental frequencies and hence concluded that the nonlinear components of the wavefield were produced not in the subsurface, but by the sources themselves. Campman et al. [18] also argued that the nonlinear wavefield observed in [3] was carried by surface waves and was generated in the shallow subsurface. However, Campman et al. [18] recorded the data only at ten levels in the well. The data points showing the ratio of the amplitudes of the second harmonic to the fundamental frequency against depth in the well were sparse, and hence these results are somewhat inconclusive.

This limitation can be overcome by using downhole fiber-optic distributed acoustic sensing (DAS) [19,20,21]. A fiber-optic DAS cable can be permanently deployed in a borehole, enabling positioning of seismic receivers in the subsurface along the full extent of a well. Importantly, DAS is much more sensitive than geophones, which can help to record and analyze weak nonlinear signals. Additionally, unlike downhole geophones, DAS is free of any mechanical components and hence is much less likely to cause any nonlinear effects on the receiver end.

We conducted two field experiments with downhole DAS at two different sites using different types of seismic sources: (1) two vibroseis sources generating signals with different monochromatic frequencies; (2) a surface orbital vibrator (SOV) with two eccentric motors working at different frequencies. In these experiments, we analyzed the nonlinear components of the wavefield recorded along the boreholes, demonstrating that they were generated in rocks at depth and were carried by body waves. Additionally, we leveraged the recent developments in laboratory methods of measurements of elastic properties of rocks at seismic frequencies and conducted laboratory tests on the effect of saturation on the nonlinear components of the wavefield in a sandstone. We observed that substitution of a saturating fluid in a sandstone sample affected second harmonics and combinational frequencies. These results form a basis for further research and demonstrate how the current state of technology can further advance development of reservoir monitoring and characterization methods using nonlinear seismic effects.

## 2. Nonlinear Elasticity

Seismic exploration studies the subsurface by using elastic waves, which are usually generated by an active seismic source, but can also be produced by earthquakes, oceanic microseisms, or anthropogenic noise. The behavior of elastic waves is described by the wave equation, which originates from Newton’s second law and Hooke’s law:

(1) $$\nabla \cdot \sigma =\rho \ddot{u}\;\mathrm{and}\;\sigma =C\varepsilon ,\;$$

where σ is the stress tensor, ρ is the density, $\ddot{u}$ is the second-order time derivative of the displacement vector, C is the stiffness tensor, and ε is the strain tensor. Components of the strain tensor can be expressed via the displacement as

(2) $$\varepsilon_{ij}=\frac{1}{2}\left(\frac{\partial u_{i}}{\partial x_{j}}+\frac{\partial u_{j}}{\partial x_{i}}+\frac{\partial u_{k}}{\partial x_{i}}\frac{\partial u_{k}}{\partial x_{j}}\right).$$

In linear elasticity, the product of partial derivatives of displacement in Equation (2) is assumed to be negligible. Keeping this term while substituting Equation (2) into the system of Equation (1) introduces nonlinear terms, which are usually referred to as geometrical nonlinearity. Its effect is often negligibly small compared to the nonlinearity of the relation between stress and strain (known as physical nonlinearity), which depends on composition, saturation, porosity, and other properties of the medium. Taking physical nonlinearity into account alters Hooke’s law:

(3) $$\sigma_{ij}=C_{ijkl}\varepsilon_{kl}+\sigma_{ij}^{n},$$

where $\sigma_{ij}^{n}$ includes the nonlinear terms. Substitution of Equation (2) into the right-hand side of Equation (3) yields

(4) $$\sigma_{ij}=C_{ijkl}\frac{\partial u_{k}}{\partial x_{l}}+M_{ijklmn}\frac{\partial u_{k}}{\partial x_{l}}\frac{\partial u_{m}}{\partial x_{n}}+\cdots .$$

Here, $M_{ijklmn}$ is the sixth rank tensor, which relates second-order spatial derivatives of displacement to stress. Substituting this nonlinear Hooke’s law into Newton’s second law yields the generalized wave equation. For example, neglecting the third- and higher-order components in Equation (4) and assuming an isotropic medium yields

(5) $$\begin{array}{l} \rho \frac{\partial^{2}u_{i}}{\partial t^{2}}-\mu \frac{\partial^{2}u_{i}}{\partial x_{k}\partial x_{k}}-\left(K+\frac{\mu }{3}\right)\frac{\partial^{2}u_{l}}{\partial x_{l}\partial x_{i}}\\ \;=\left(\mu +\frac{A}{4}\right)\left(\frac{\partial^{2}u_{l}}{\partial x_{k}\partial x_{k}}\frac{\partial u_{l}}{\partial x_{i}}+\frac{\partial^{2}u_{l}}{\partial x_{k}\partial x_{k}}\frac{\partial u_{i}}{\partial x_{l}}+\frac{\partial^{2}u_{i}}{\partial x_{l}\partial x_{k}}\frac{\partial u_{l}}{\partial x_{k}}\right)\\ \;+\left(K+\frac{\mu }{3}+\frac{A}{4}+B\right)\left(\frac{\partial^{2}u_{l}}{\partial x_{i}\partial x_{k}}\frac{\partial u_{l}}{\partial x_{k}}+\frac{\partial^{2}u_{k}}{\partial x_{l}\partial x_{k}}\frac{\partial u_{i}}{\partial x_{l}}\right)+\left(K-\frac{2}{3\mu }+B\right)\left(\frac{\partial^{2}u_{i}}{\partial x_{k}\partial x_{k}}\frac{\partial u_{l}}{\partial x_{l}}\right)\\ \;+\left(\frac{A}{4}+B\right)\left(\frac{\partial^{2}u_{k}}{\partial x_{l}\partial x_{k}}\frac{\partial u_{l}}{\partial x_{i}}+\frac{\partial^{2}u_{l}}{\partial x_{i}\partial x_{k}}\frac{\partial u_{k}}{\partial x_{l}}\right)+\left(B+2C\right)\left(\frac{\partial^{2}u_{k}}{\partial x_{i}\partial x_{k}}\frac{\partial u_{l}}{\partial x_{l}}\right) \end{array}$$

where A, B, and C are the third-order elastic constants defining the nonlinearity of the stress–strain relationship. Note that the right-hand side of Equation (5) contains the same second spatial derivatives of displacement as present in the left-hand side but multiplied by components of strain $\frac{\partial u_{l}}{\partial x_{i}}$. Away from seismic sources, strain amplitudes are usually very small (smaller than 10^−6^). If nonlinear constants A, B, and C are zero, there is no physical nonlinearity in the medium and only geometrical nonlinearity is present, but then all the terms in the right-hand side of Equation (5) are negligible and the medium can be treated as linear. Thus, the effect of nonlinearity is significant only when A,B,C≫K,μ. For rocks, A, B, and C are typically larger than 10^12^ Pa [22,23].

Another source of nonlinearity is the distortion of the source signature due to mechanical interaction of the source mechanical components with the surface and with each other. Such nonlinearity is called mechanical and usually results in generation of harmonics in the emitted signal. For example, this effect is typical for modern seismic vibrators [9].

To illustrate how nonlinearity of the earth results in generation of combinational frequencies, we can express the output of such a nonlinear system as a power series:

(6) $$F\left(x\right)=a_{0}+a_{1}x+a_{2}x^{2}+a_{3}x^{3}+\cdots ,$$

where *x* is the input signal and $a_{0}$, $a_{1}$, $a_{2}$,… are the constant parameters characterizing the nonlinear system. If the input signal is the combination of two harmonic waves $x\left(t\right)=A_{1}\cos\left(\omega_{1}t\right)+A_{2}\cos\left(\omega_{2}t\right)$, Equation (6) can be expressed as

(7) $$F\left(x\right)=a_{0}+a_{1}\left[A_{1}\cos\left(\omega_{1}t\right)+A_{2}\cos\left(\omega_{2}t\right)\right],+a_{2}{\left[A_{1}\cos\left(\omega_{1}t\right)+A_{2}\cos\left(\omega_{2}t\right)\right]}^{2}+\cdots =\cdots +\;a_{2}[\frac{A_{1}^{2}}{2}\left(1+\cos\left(2\omega_{1}t\right)\right)+A_{1}A_{2}\left[\cos(\left(\omega_{1}+\omega_{2})t\right)+\cos(\left(\omega_{1}-\omega_{2})t\right)\right]+\frac{A_{2}^{2}}{2}\left(1+\cos\left(2\omega_{2}t\right)\right)]+\cdots \;.$$

The output of this nonlinear system contains harmonics of the original frequencies $2\omega_{1}$, $2\omega_{2}$, $3\omega_{1}$, $3\omega_{2}$, etc., their combinations $\omega_{1}+\omega_{2}$ and $\omega_{1}-\omega_{2}$ and more complicated intermodulation products $N\omega_{1}+M\omega_{2}$, where *N* and *M* are integers.

## 3. Experiment 1: Vibroseis Source

**Figure 1:**
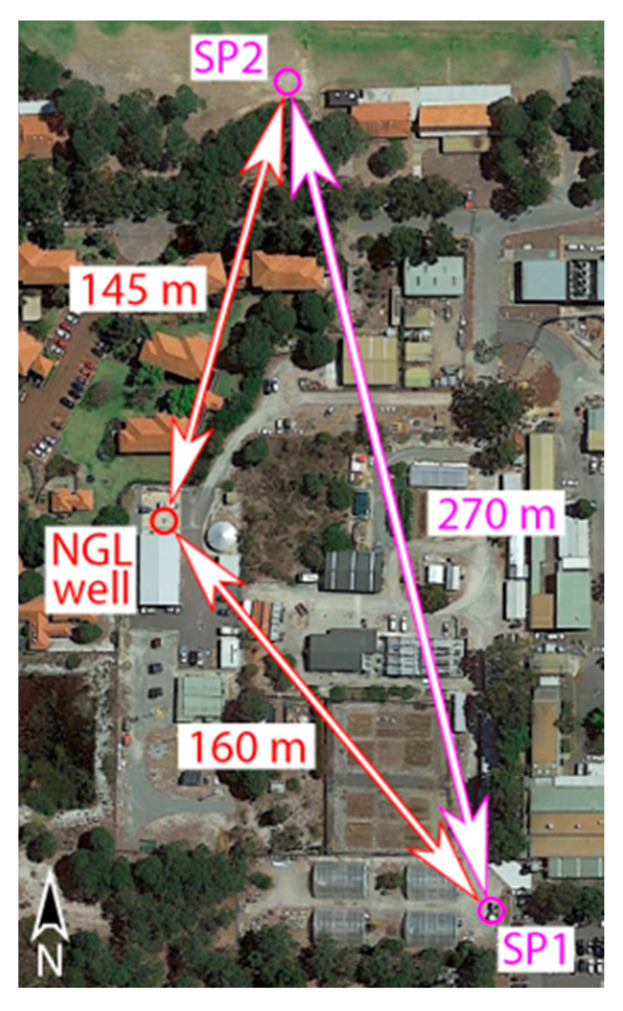
The map of the experiment with vibroseis sources at Curtin campus, with the wellhead and two source locations (SP1 and SP2) marked by circles.

At the Curtin GeoLab facility located within the main (Bentley) campus of Curtin University in Perth, Western Australia (https://ceg.curtin.edu.au/facilities/curtin-geolab/) (accessed on 29 November 2022), vibroseis sources were used to explore nonlinear seismic effects. The facility includes a training well with a ~900 m deep vertical borehole. A fiber-optic DAS cable is cemented behind the fiberglass casing along the full extent of the well, which ensures excellent coupling with the formation. The DAS cable in the well was connected to an iDASv2 interrogator unit (Silixa Ltd., Borehamwood, UK) via ~250 m of cable coiled on the surface. The seismic waves were generated by two 26,000 lbs. Inova vibroseis trucks. The vibrators were set to generate monochromatic pulses with fundamental frequencies of 30 and 22 Hz. The experiment consisted of a nearfield test, when vibrators were located next to each other at the shot point SP1, which is 160 m away from the well head; and a far field test, when the vibrators were located at two different shot points (SP1 and SP2) ~270 m apart (Figure 1). In both tests, we first operated the vibrators separately and then simultaneously, making ten 30 s shots with 0.5 s cosine tapers and 5 s listening time in-between shots. The peak force was set to 30% when the vibrators were next to each other to minimize the risk of mechanical damage of the equipment. A 70% peak force was used when the vibrators were 270 m apart.

**Figure 2:**
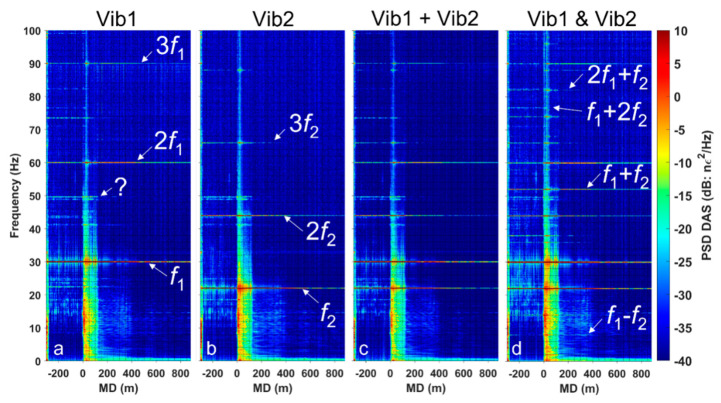
Spectrograms of the recorded data: (**a**) Vibrator 1 working solo; (**b**) Vibrator 2 working solo; (**c**) the signals from the two vibrators working solo summed up to simulate their linear superposition; (**d**) two vibrators working simultaneously. The measured depth (MD) starts at the wellhead and its negative values correspond to the part of the cable coiled around the well on the surface.

**Figure 3:**
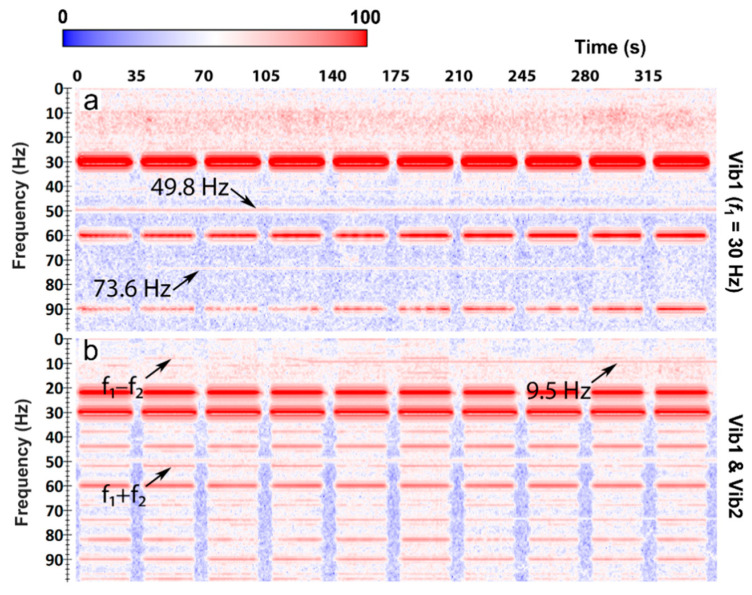
Time–frequency spectra of the data recorded by a single DAS channel in the well at a depth of ~60 m. (**a**) Vibrator 1 working solo at SP2; (**b**) two vibrators working simultaneously at SP1 and SP2.

To increase the signal-to-noise ratio, we stacked all ten shots from each firing sequence and then computed spectrograms for every channel along the fiber (Figure 2). Figure 2a,b shows that the vibrators, when working solo, generate higher harmonics of the fundamental frequencies, which are probably the result of mechanical nonlinearity of the vibrators and interaction at the near surface. The time–frequency representation of the data (Figure 3) helps to delineate the source-generated signal from the ambient human-related noise. Note that the frequencies generated by the source are discontinuous on the record because of the 5 s time gap between shots. At the same time, some components of the wavefield, such as 9.5, 49.8, and 73.6 Hz, are present even between shots, indicating that they are not related to active seismic sources. For example, Figure 3b shows that the 9.5 Hz signal emerges in the middle of the vibrators’ firing sequence and is carried through till the end of the shooting.

**Figure 4:**
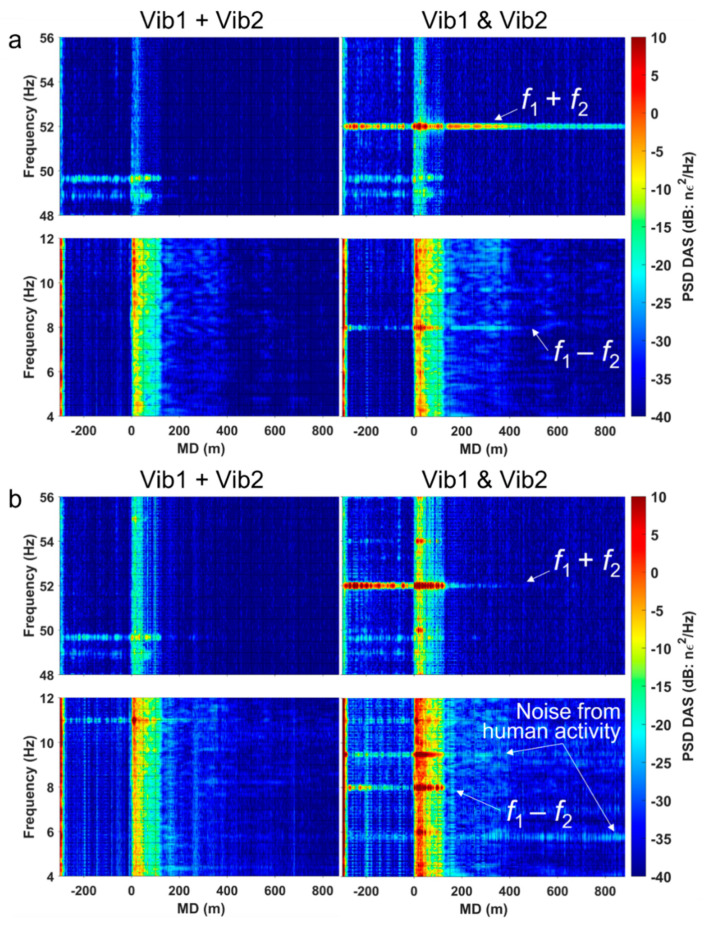
Spectrograms of the recorded data during (**a**) nearfield test and (**b**) far-field test zoomed in around combinational frequencies of 8 Hz and 52 Hz. Images in the left column show the spectrogram of the linear superposition of the data recorded when the vibrators were working solo; images in the right column show the spectrogram of data recorded when two vibrators were working simultaneously. The measured depth (MD) starts at the wellhead and its negative values correspond to the part of the cable coiled around the well on the surface.

Comparison of the spectrograms of the data recorded when two vibrators were working at the same time (Figure 2d) against the linear superposition of the two signals (Figure 2c) gives a clear indication of the presence of nonlinear effects in the earth. Simultaneous operation of the two vibrators produces combinational and intermodulation frequencies, which are not present in the linear superposition of the two signals. Combinational frequencies f_1_ − f_2_ = 8 Hz and f_1_ + f_2_ = 52 Hz are seen at the surface and can be traced to deeper sections in the well (Figure 4). In the nearfield test, the combinational 52 Hz frequency signal is seen at the bottom of the well (at a depth of 900 m). Therefore, the energy generated by a nonlinear medium propagates not only as surface waves, but as body waves as well. This can be further confirmed by observing a broadband DAS VSP seismogram from the same well and using the same acquisition geometry [24]; this seismogram shows that the wavefield is dominated by P and S waves, and exhibits no surface waves or borehole modes. Additionally, the tests suggest that nonlinear seismic effects do originate in rocks because the mechanical nonlinear interaction of the vibrators was excluded in the far-field test. Delineation of the exact location of the source of nonlinearity requires further analysis.

**Figure 5:**
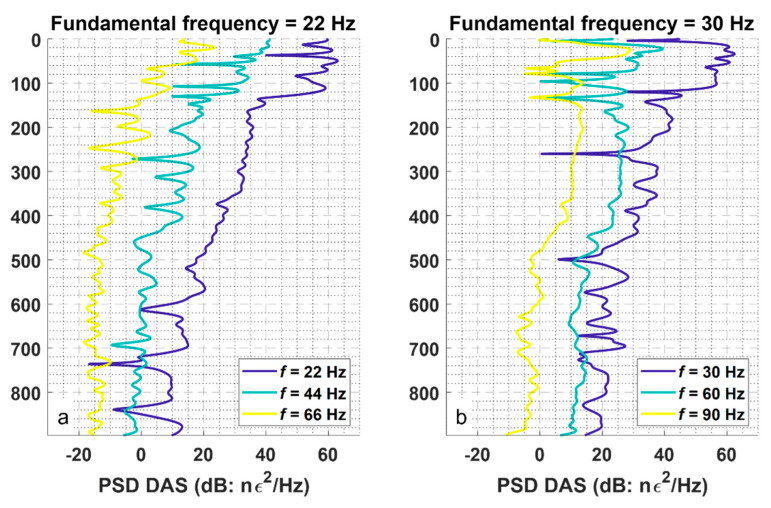
(**a**) Power spectral densities of the signals carried as the fundamental frequency of 22 Hz and its higher harmonics 44 and 66 Hz along the well. The data recorded when the first vibrator was working solo at SP1. (**b**) Power spectral densities of the signals carried as the fundamental frequency of 30 Hz and its higher harmonics 60 and 90 Hz along the well. The data was recorded when the first vibrator was working solo at SP2.

Analysis of the amplitudes of the nonlinear components of the wavefield recorded in the well provides an insight into nonlinear seismic phenomena. Figure 5 shows the power spectral densities of both fundamental frequencies of 22 and 30 Hz and their harmonics recorded along the well when only one vibrator was operating. As expected, the amplitudes of all three components decrease with increasing depth of the receiver. However, the fundamental components decay somewhat faster than the second harmonics (44 and 60 Hz). This suggests that nonlinear components of the wavefield are generated or reinforced in the subsurface. Had the harmonics been produced solely by the source at the surface, we would expect to see faster attenuation of the higher frequency signal as reported by Campman, et al. [18], which is not the case in our data.

## 4. Experiment 2: Permanent Surface Orbital Vibrators

**Figure 6:**
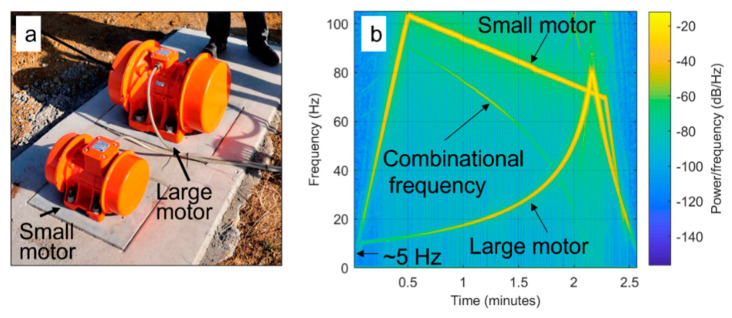
(**a**) Surface orbital vibrator (SOV) installed at the Otway project site. (**b**) Spectrogram of the signal recorded by the reference 3C geophone buried 3 m below the SOV during the production sweep.

The second field experiment was conducted at the CO2CRC Otway site in the Australian state of Victoria (https://co2crc.com.au/research/otway-international-test-centre/) (accessed on 20 November 2022). The Otway site is a designated area for development and testing of technologies for carbon capture and storage. The site contains a continuous seismic monitoring system, which includes five monitoring wells instrumented with DAS and nine permanent surface orbital vibrators (SOVs) operating daily [25]. The purpose of the system is monitoring injection and migration of CO_2_ in the subsurface [26]. For this experiment, we used two elements of the monitoring system: a DAS cable installed behind the casing of one of the monitoring wells and an SOV deployed on the surface ~40 m away from the wellhead. The SOV has two eccentric motors of different sizes (Figure 6a), which can generate sweeps independently from each other [27]. SOVs typically operate every second day for 2.5 h starting with a 20 min warmup sequence, where the two motors make a series of rotations at quasi-monochromatic frequencies of f_1_ = 60 Hz (large motor) and f_2_ = 80 Hz (small motor), followed by the production sequence, where the motors generate sweep seismic signals within a 10–105 Hz frequency band. The generated wavefield is continuously recorded by the DAS system.

Another component of the recording system is a reference geophone buried 3 m beneath the SOV. The spectrogram of the wavefield recorded during the production sequence is shown in Figure 6b. The small motor produces a linear downsweep from 105 to 70 Hz, while the large motor produces a quadratic upsweep from 10 to 80 Hz. The reference geophone recorded these two fundamental signals and the generated combinational signal. The presence of this combinational frequency is an indicator of nonlinear interaction of the sweeps produced by the two motors; however, it is unclear from these data alone whether it is caused by mechanical interaction of the motors or the physical nonlinearity of the earth.

**Figure 7:**
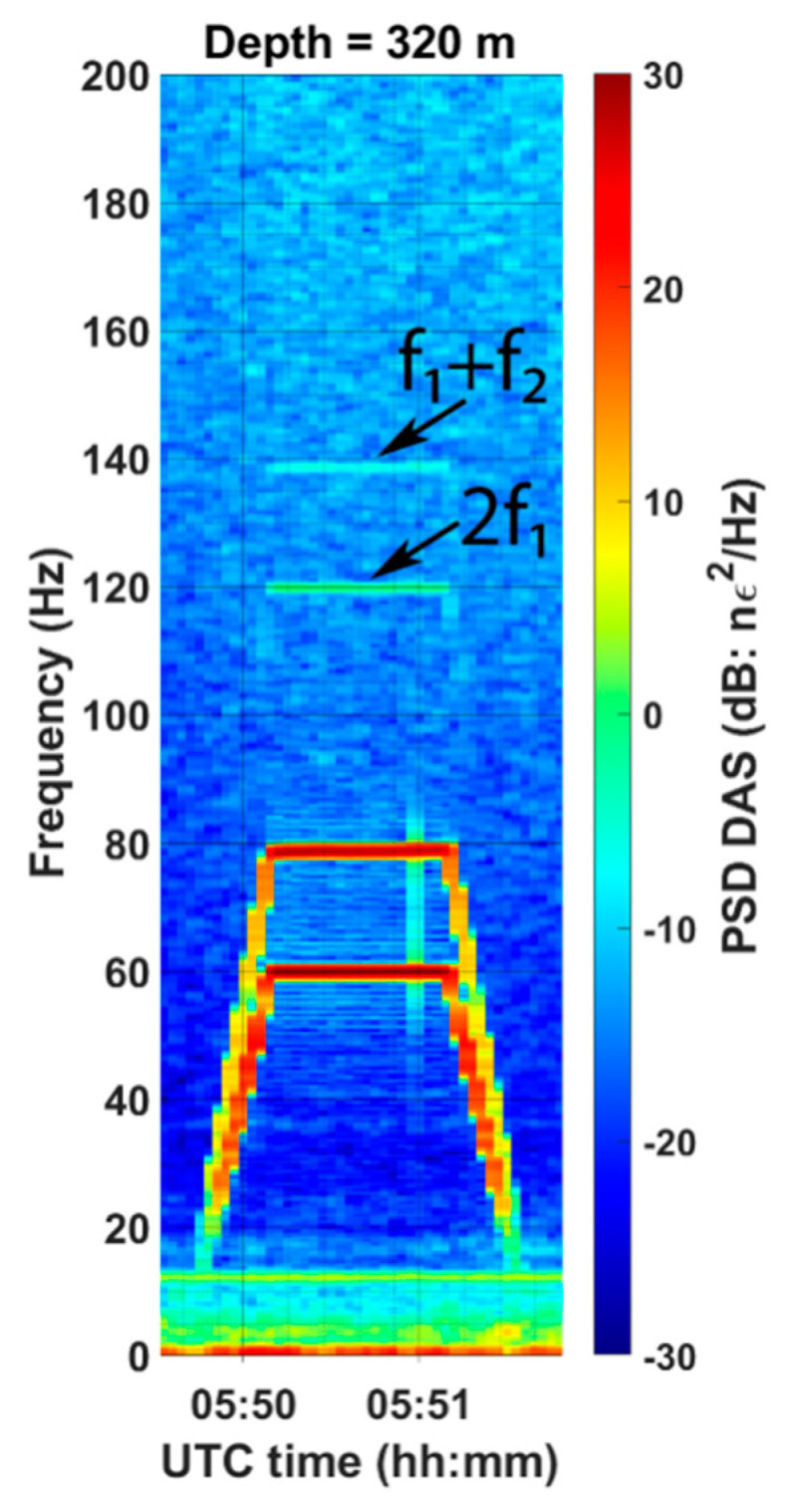
Spectrogram of the signal recorded 320 m deep in the well during the warmup sequence of the SOV by DAS.

**Figure 8:**
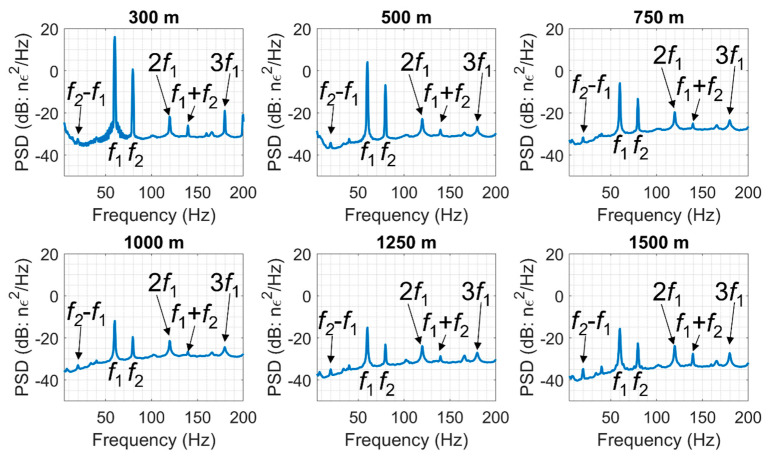
Power spectral densities of the DAS signals recorded 300, 500, 750, 1000, 1250, and 1500 m deep in the well during the warmup sequences of the SOV. Fifty sweeps were stacked together to increase the signal-to-noise ratio.

To clarify this question, we analyzed the DAS data looking for combinational frequencies. Figure 7 shows the power spectral density of the wavefield recorded at a depth of 320 m during the warmup sequence of the SOV. At this depth, we detected the combinational frequency *f*_1_ + *f*_2_ of the two warmup sweeps confirming that the energy carrying such frequencies propagates in the form of body waves. Figure 8 shows the power spectral density of the signal recorded at six different levels in the well (fifty sweeps were stacked together to increase the signal-to-noise ratio). In addition to the fundamental frequencies gradually decaying with depth, the data show combinational frequencies *f*_1_ − *f*_2_ and *f*_1_ + *f*_2_ at all levels in the well and harmonics of the 60 Hz signal. The harmonics of the 80 Hz signal are weak and barely detectable in the well as they are produced by the small motor, which transmits less energy into the ground compared to the large motor.

The data also show 3*f*_1_ = 180 Hz and *f*_1_ + *f*_2_ = 140 Hz components of the wavefield as deep as 1500 m, which would have been unlikely had they been generated on the surface only. Additionally, these frequencies appear to decay slower than the fundamental frequencies generated by the motors on the surface. These observations suggest that the nonlinear interaction of seismic waves occurs not only on the surface, but within the subsurface as well.

## 5. Experiment 3: Laboratory Tests

If the nonlinear elastic effects observed in the field are caused by the physical nonlinearity of rocks, they should also be evident in laboratory tests. Laboratory tests provide an opportunity not only to corroborate the existence of nonlinear effects under controlled conditions, but also to explore their dependence on various environmental conditions such pressure or degree of saturation [28]. This is indeed the case; there exists substantial evidence of nonlinear elasticity of rocks in a vast range of frequencies from quasistatic to ultrasonic [12,13,29]. However, while at ultrasonic frequencies the nonlinear effects have been observed at very small strain amplitudes (<10^−6^), quasistatic tests at seismic frequencies are limited to relatively large strains (>10^−4^) [29]. 

**Figure 9:**
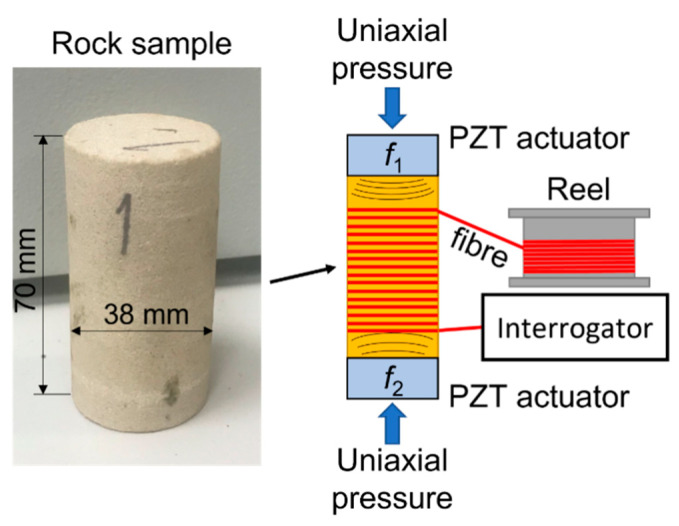
Schematic of the laboratory setup.

To better explore nonlinear seismic phenomena in direct stress-strain tests, we conducted laboratory experiments using a modified forced-oscillation stress-strain method and apparatus developed at Curtin University [30,31]. In the original setup, a sample is placed inside a stainless-steel rig, where the confining pressure can be delivered to the sample via a hydraulic actuator and a triaxial core holder. The sample is subjected to a periodic sinusoidal load in a frequency range from 0.01 to 400 Hz applied via a piezoelectric stack actuator mounted at the top of the assembly. The induced periodic load causes deformation of a sample, which is captured by strain sensors. The modifications to the method and apparatus done in this work included introduction of a fiber optic DAS cable for strain measurements [32] and the second piezoelectric actuator for generating two different monochromatic signals at the same time. The schematic of the experimental setup is shown in Figure 9.

**Figure 10:**
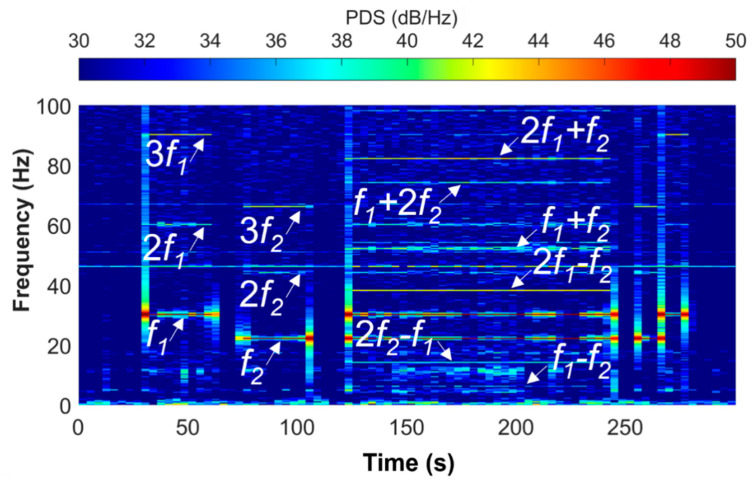
Spectrogram (power spectral density) of the signal recorded in the middle of the Bentheimer sandstone sample during the laboratory test. Fundamental frequencies f1 and f2, their harmonics and combinational products are annotated.

For the laboratory tests, we used a Bentheimer sandstone sample [33,34], which typically contains 90–95 wt% of quartz and <3 wt% of clays, has a porosity of 21–27% and a permeability of 0.5–3.0 Darcy. The sample was placed between two piezoelectric transducers and the fiber optic cable was wound around the sample for measuring the radial component of the strain induced by periodic oscillations of the transducers. Like in the field experiments with vibroseis sources, we set the actuators to work at two fundamental frequencies of 30 and 22 Hz and did the tests when two actuators were working independently and simultaneously. Table 1 shows the protocol of the experiment, including the list of frequencies and the duration of each stage of the tests. The spectrogram of the signal recorded in the middle of the sample is shown in Figure 10. First, one transducer was operating at 30 Hz for about 40 s. The signal response in the sample contains this fundamental frequency and its higher harmonics (60 and 90 Hz). Then, the second transducer was working solo at 22 Hz frequency. Again, this fundamental frequency and its higher harmonics (44 and 66 Hz) were recorded on the fiber. Then, the signal recorded when the two transducers were working at the same time contains both fundamental frequencies, their higher harmonics, as well as combinational and intermodulation frequencies. Several frequencies (46, 50 and 67 Hz) are present throughout the whole experiment including the time between different stages, when none of the transducers were working. These frequencies originate from the laboratory noise, which may include electrical currents, an air conditioning system and other laboratory equipment operating at the time of the tests.

**Figure 11:**
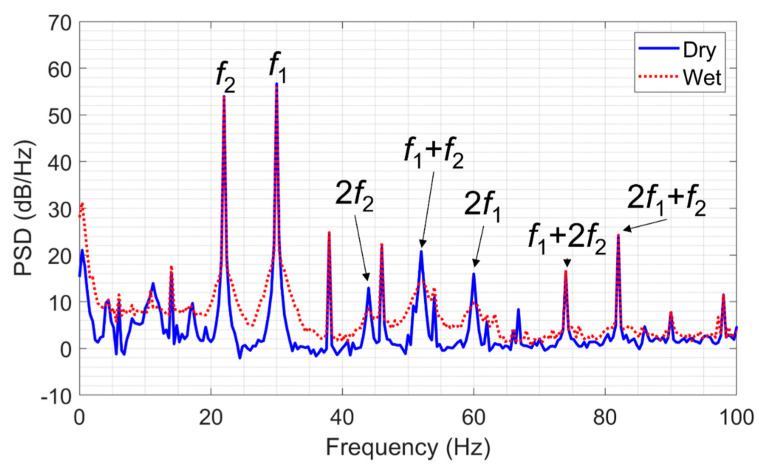
Power spectral densities of the signals recorded during laboratory tests on the sandstone sample in a dry and wet states.

After performing the measurements on the dry sample, we partially saturated the sample with water and repeated the tests. Figure 11 shows a comparison of the power spectral densities of the signals recorded in the middle of the sample in both dry and wet states. Saturation of the sample with water decreases the power spectral density of the following frequency components: 2*f*_1_, 2*f*_2_ and *f*_1_ + *f*_2_, but does not affect the amplitude of other intermodulation components. This result confirms that as fluid substitution in rocks changes their elastic properties, it also affects the nonlinear seismic response. This phenomenon can potentially be used for subsurface characterization but requires further study.

## 6. Discussion

Our field experiments suggest that nonlinear components of the wavefield are generated in rocks and propagate as body waves. While prior field investigations in nonlinear seismology used surface reflection methods, our experiments using VSP acquisition geometry allowed for recording of direct transmitted energy. Recording direct arrivals at multiple levels offers a more accurate assessment of the seismic wavefield in the rock as it progresses downward. This is a critical development as downhole receivers provide additional information about the propagation and amplitudes of the nonlinear components. This paper is focused on the evidence of nonlinear effects in field and lab experiments. Detailed analysis of amplitude and phase characteristics of nonlinear components will be a subject of future studies.

Another crucial element of our experiments was the use of fiber-optic DAS as a seismic receiver. DAS is better suited for studying nonlinear seismic effects than standard borehole geophone tools. First, DAS can be permanently deployed in a well enabling simultaneous measurements along the full extent of the borehole, which realistically cannot be achieved with geophones. Second, a geophone is an electro-mechanical tool, which can become a nonlinear mixer for seismic waves, contributing to generation of the combinational and intermodulation frequencies. In contrast, DAS is free from any mechanical components, and is unlikely to be a source of significant nonlinearity for seismic waves.

For that reason, we used DAS in the laboratory experiments as well. Our laboratory tests are quasistatic in nature, because the wavelengths in rocks at seismic frequencies are significantly larger than the size of our experimental setup. Hence, our laboratory experiments did not involve wave phenomenon as such. Nonetheless, these experiments did show the existence of combinational and intermodulation frequencies in the sample during the tests and demonstrated that the saturating fluid may reduce the nonlinear elastic response in rocks. This could possibly be explained by increased adhesion between adjacent grains [35]. Further research is required to establish the relationship between the composition of saturated rocks and their nonlinear elastic properties.

Nonlinear responses of rocks to different saturating fluids can be potentially useful for subsurface fluid characterization, including petroleum exploration and monitoring of hydrocarbon production or CO_2_ geosequestration. Nonlinear seismic effects can also be used to broaden the frequency band of seismic sources, which is especially important for SOVs, because the sweep signal that an SOV produces lacks low frequencies (its amplitude scales with the frequency squared). Thus, producing a difference frequency using two simultaneous high-frequency sweeps may be more beneficial than using an SOV at low frequencies directly. Practical applications of nonlinear seismic effects are not limited to exploration and production only. These effects can be potentially used for safety monitoring of civil engineering infrastructures (e.g., bridges, tunnels, dams, etc.) using nonlinear interaction of elastic waves with defects [36]. Additionally, these applications can use various seismic sources including ambient seismic energy propagating in the earth. In our experiments, we used two active seismic sources, but it may be possible to use earthquakes or unrelated human activity to modulate a source signal or completely avoid using any active source if these ambient sources are sufficiently strong. Indeed, the nonlinear effects of soil layers on ground motion induced by earthquakes are well known in engineering seismology [37,38,39].

## 7. Conclusions

The field experiments at two different sites demonstrated that seismic waves emitted by two seismic sources on the surface generate combinational and intermodulation frequencies. These nonlinear components of the wavefield were recorded within the subsurface, confirming that they are carried by body waves. Furthermore, the analysis of amplitudes of the nonlinear and fundamental frequencies suggests that nonlinear components of the wavefield are generated or reinforced in the subsurface.

To support the observation of the field experiments, we have developed a method for laboratory measurements of the effect of fluid substitution on the nonlinear components of the wavefield. To this end, we have modified a forced-oscillation apparatus introducing two sources of oscillations working at different frequencies. We observed that saturating a dry sandstone sample with water affects the amplitudes of nonlinear products of the fundamental frequencies. The observations from both laboratory and field experiments confirm the potential of using the nonlinear seismic effects for reservoir fluid characterization.

Fiber-optic DAS was a crucial component of both field and laboratory experiments. Fiber-optic DAS is free of any mechanical parts and hence should not cause the nonlinear interaction of waves by itself, and it is more sensitive than the standard strain gauges used in the laboratory. For field measurements, DAS can be permanently and cost-efficiently deployed in the subsurface providing simultaneous measurements of the wavefield along the full extent of a well inside rock formations. 

Although the field of nonlinear seismology is in its infancy, and practical applications have yet to be developed, our work demonstrated that there are adequate tools to detect and analyze nonlinear seismic effects. Borehole DAS enables detection of nonlinear seismic effects in rock formations, and our laboratory equipment provides an opportunity to study this phenomenon in a controlled environment.

## Figures and Tables

**Table 1:** Protocol of the laboratory experiment.

Stage No.	Frequencies (Hz)	Duration (s)
1	30	40
2	22	40
3	30 and 22	120
4	22	20
5	30	20

## Data Availability

The data used in the experiment 1 and experiment 3 are available at the shared repository (https://doi.org/10.6084/m9.figshare.19027760.v1 (accessed on 30 November 2022)). The data used in the experiment 2 is not publicly available.
